# Iatrogenic iron overload caused by iron isomaltoside treatment: A case report

**DOI:** 10.1097/MD.0000000000047000

**Published:** 2026-01-09

**Authors:** Yao Hu, Min Sun, Lihui Zhong, Dan Luo, Yuxi Wu, Yuehua Yan, Yuanbing Xiang

**Affiliations:** a Department of Nephrology, Affiliated Hospital & Clinical Medical College of Chengdu University, Chengdu, Sichuan Province, China; b Department of Urology Division, West China Hospital, West China School of Medicine, Sichuan University, Urology Research Institute, Chengdu, China.

**Keywords:** anemia, iatrogenic iron overload, intravenous iron therapy, iron isomaltoside, liver iron concentration

## Abstract

**Rationale:**

Iron-deficiency anemia is commonly treated with intravenous (IV) iron supplementation; however, excessive use can lead to iron overload and subsequent organ damage. Iron isomaltoside, a newer IV iron formulation, may also contribute to iron overload, necessitating vigilant monitoring by clinicians. This report presents a case of iatrogenic iron overload induced by iron isomaltoside therapy. To the best of our knowledge, this is the first documented case of iron overload associated with iron isomaltoside.

**Patient concerns:**

A 65-year-old female patient with systemic lupus erythematosus developed iron overload after receiving 7500 mg of iron isomaltoside over 5 weeks. Laboratory results showed significantly elevated serum ferritin levels (4336.47 ng/mL), and magnetic resonance imaging confirmed iron deposition in both the liver and spleen.

**Diagnoses:**

Laboratory tests and magnetic resonance imaging confirmed iron overload.

**Interventions:**

The patient was treated with deferasirox for iron chelation.

**Outcomes:**

After 8 months of iron removal treatment, the patient’s serum ferritin level gradually decreased to 1236.2 ng/mL, accompanied by improvements in hyperpigmentation and fatigue. No severe adverse events or gastrointestinal symptoms were observed during deferasirox administration, and kidney function remained stable throughout.

**Lessons:**

This case highlights the risk of iron overload associated with unmonitored IV iron supplementation. Monitoring iron levels is crucial to prevent complications, particularly in high-risk patients. Iatrogenic iron overload can arise from excessive IV iron isomaltoside administration, emphasizing the importance of vigilant monitoring of iron metabolism to prevent adverse outcomes.

## 1. Introduction

Iron deficiency is a primary cause of anemia worldwide, significantly affecting global health by contributing to disability and mortality, particularly among vulnerable populations.^[1]^ Iron-deficiency anemia (IDA) is a hypoproliferative microcytic anemia caused by iron deficiency, which impairs hemoglobin synthesis and erythropoiesis. It is commonly associated with chronic kidney disease, gastrointestinal disorders, reproductive health issues in women, nutritional deficiencies in children, and malignancies. IDA is strongly associated with several chronic conditions, including chronic kidney disease, gastrointestinal disorders, health issues affecting women and children, and certain cancers. The treatment of IDA typically involves 3 main approaches: oral iron supplements, intravenous (IV) iron therapy, and blood transfusion, with IV iron therapy being commonly used in clinical practice. Iron isomaltoside is an IV iron formulation that, compared to sucrose iron, more rapidly and significantly improves hematological parameters and alleviates fatigue. Both preparations exhibit similar safety profiles, with low rates of hypersensitivity reactions, cardiovascular events, and severe adverse effects.^[2]^ However, because the body lacks a dedicated organ for iron excretion, iron is primarily eliminated through epithelial shedding and bleeding.^[3,4]^ Excessive iron supplementation can lead to iron overload, a systemic disorder characterized by the accumulation of iron in various tissues, resulting in structural damage and dysfunction of vital organs, including the liver, heart, and endocrine glands. This condition may arise from genetic mutations, such as those affecting the high-iron gene, or from secondary factors, such as chronic blood transfusions or the inappropriate use of iron supplements. This report presents a case of iron overload in a patient with IDA who received multiple IV iron infusions.

## 2. Ethical approval

Written informed consent for publication was obtained from the patient. As a retrospective case report, this study was exempt from ethical review by the Ethics Committee of the Affiliated Hospital of Chengdu University.

## 3. Case presentation

A 65-year-old female, diagnosed with systemic lupus erythematosus over 10 years ago, has been undergoing long-term treatment with hydroxychloroquine, methylprednisolone, and other medications. She presented to our department on January 16, 2025, with a 2-month history of skin color changes and a 5-day history of coughing. Her medical history included chronic gastritis, osteoporosis, femoral head necrosis, and laparoscopic closure of distal sigmoid colostomy.

Physical examination revealed a blood pressure of 113/52 mm Hg, a weight of 55 kg, and clear consciousness. Additionally, the patient exhibited generalized skin darkening with a bronze-like, dull complexion and no abnormal lung sounds. No abnormal pulsations were detected in the precordial region, and the heart size was normal. The heart rate was 97 beats/min with a regular rhythm, and no pathological murmurs were auscultated in any of the valve areas. The abdomen was soft, with a left-sided colostomy, negative Murphy sign, no tenderness over the liver or kidney areas, no lower extremity swelling, and normal muscle strength in the limbs.

**Figure 1.**
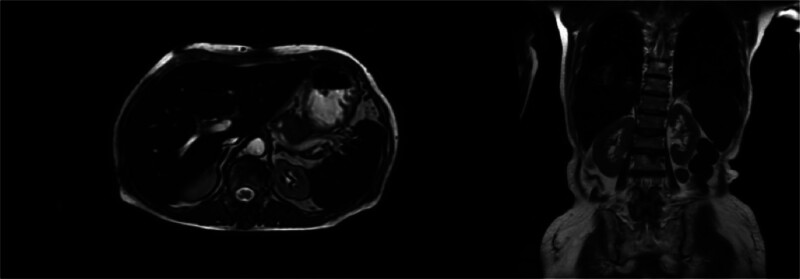
MRI examination results. MRI showing hypointense signals in the liver and spleen consistent with iron overload. MRI = magnetic resonance imaging.

Laboratory results on admission showed alanine transaminase 60.8 U/L, aspartate transaminase 42 U/L, and serum ferritin (SF) 4336.47 ng/mL. Upper abdominal magnetic resonance imaging (MRI) indicated significantly decreased T1WI and T2WI signals in the liver and spleen, which were lower than the muscle signal at the same level, suggesting iron overload (Fig. 1). Blood gas analysis, serum iron and glucose levels, kidney function, hemoglobin levels, white blood cell count, platelet count, and complement levels were all within normal ranges. Tests for hepatitis A, B, and C antibodies were negative.

Upon reviewing the patient’s history, the SF was 61.97 ng/mL in a test conducted on June 12, 2024. In September 2024, the patient underwent partial small bowel resection at the Department of Gastrointestinal Surgery. Hemoglobin was measured at 83 g/L, suggesting IDA, although iron metabolism indices were not assessed. Considering that oral iron supplementation may be less effective after small bowel surgery, the patient received IV iron therapy with iron isomaltoside at a dose of 500 mg 3 times per week for 5 weeks, for a cumulative total of 7500 mg. Ferritin and transferrin saturation were not monitored during the administration of iron isomaltoside. Since then, no iron supplements have been used, including oral forms.

**Figure 2.**
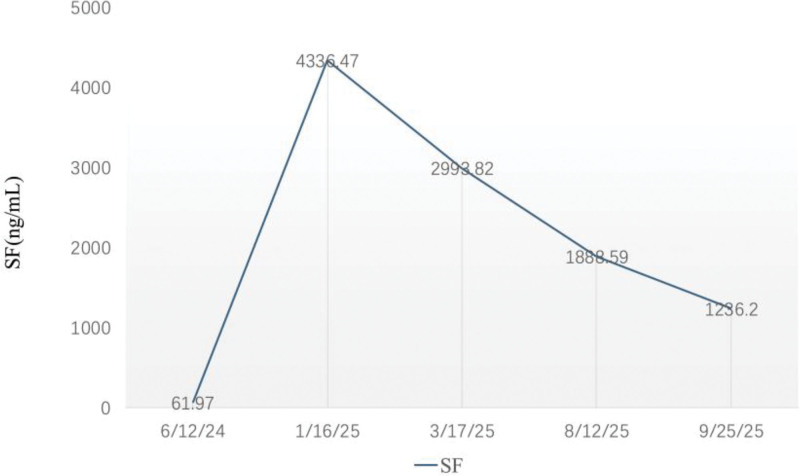
Changes in the patient’s SF. SF = serum ferritin.

**Figure 3.**
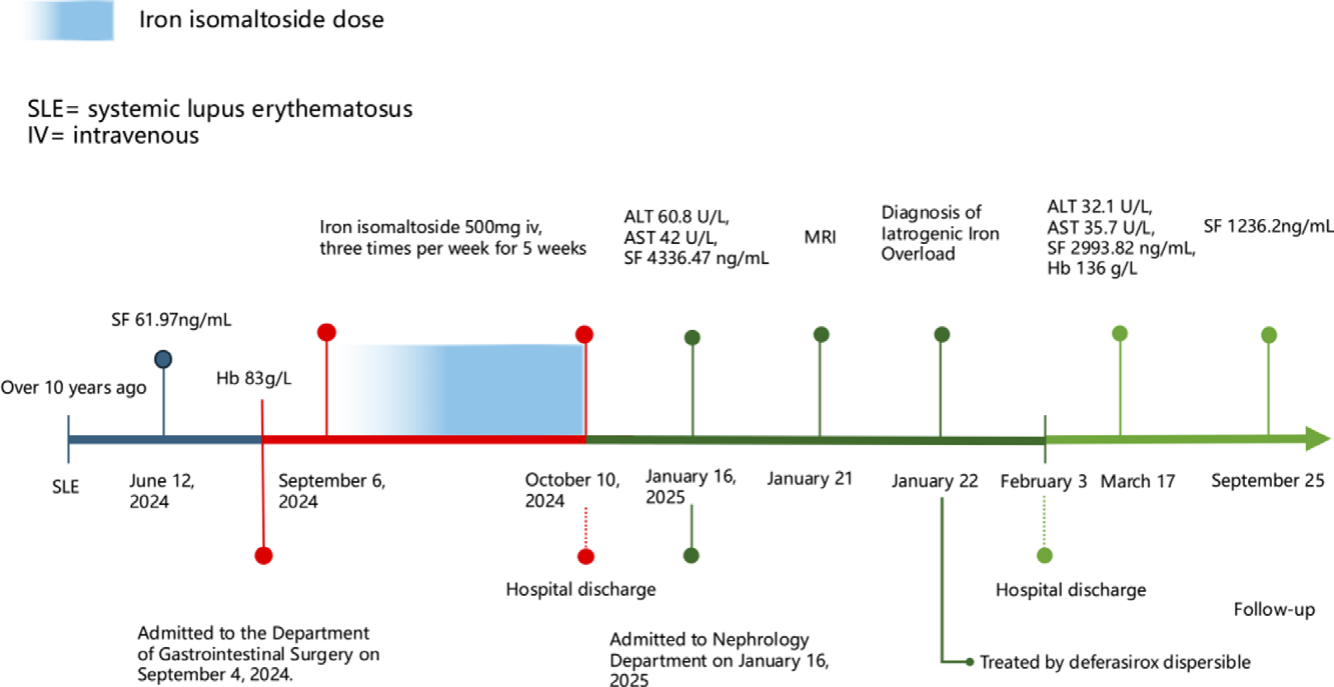
Chronological timeline. ALT = alanine transaminase, AST = aspartate transaminase, IV = intravenous, MRI = magnetic resonance imaging, SF = serum ferritin, SLE = systemic lupus erythematosus.

Upon admission, iron overload was confirmed, with no evidence of cardiac or endocrine complications, and deferasirox dispersible tablets were prescribed for iron chelation. By March 17, SF had decreased to 2993.82 ng/mL, hemoglobin had increased to 136 g/L, and alanine transaminase and aspartate transaminase levels had decreased to 32.1 U/L and 35.7 U/L, respectively. On September 25, SF decreased further to 1236.2 ng/mL (Fig. 2). During deferasirox administration, no serious adverse events or gastrointestinal symptoms were observed, and kidney function remained stable. The patient’s hyperpigmentation and fatigue improved, and clinical follow-up remains in progress. A graphical timeline of case evolution is presented in Figure 3.

This study adheres to the Case Report guidelines,^[5]^ and all patient information was de-identified. The patient provided informed consent for the publication of the case report and associated images.

## 4. Patient perspective

Our patient reported the following about her experience: “Throughout the clinical diagnosis and treatment process, I was made aware of the uncertainties and concerns surrounding the evaluation and management of my condition. At one point, I suspected that the changes in my complexion were linked to excessive medication administered during gastrointestinal surgery. However, after consulting with a nephrologist and receiving appropriate treatment, I am confident of my recovery.”

## 5. Discussion

Iron is an essential nutrient that plays a crucial role in various physiological processes, including oxygen transport, DNA synthesis and repair, and cellular metabolism. However, the human body lacks an effective physiological mechanism for iron excretion, with only approximately 1 to 2 mg lost daily through sloughing of gastrointestinal mucosal cells.^[6]^ Excess free iron ions can catalyze the formation of harmful free radicals, leading to oxidative damage. Iron overload, as observed in conditions such as hemochromatosis, can cause severe organ damage. In the current case, significant iron deposition was observed in both the liver and the spleen. The liver is the primary target organ for iron overload, and notable iron accumulation led to liver dysfunction in this patient. During iron overload, SF and hemosiderin bind excess iron, which accumulates in the liver and induces degenerative changes in liver cells. In cases of long-term excessive iron accumulation, the risk of cirrhosis and hepatocellular carcinoma increases, especially in individuals with a genetic predisposition.^[7]^ Iron overload is also strongly associated with cardiovascular diseases, contributing to myocardial injury in patients with heart failure and influencing the pathophysiology of myocardial infarction.^[8]^ Moreover, iron plays a critical role in bacterial growth and virulence, and excessive IV iron administration can exacerbate the risk and severity of infections.^[9]^

The diagnostic criteria for iron overload were established by excluding conditions such as liver disease, active inflammation, tumors, and hemolysis, with an SF level exceeding 1000 ng/mL.^[10–12]^ In this case, the diagnosis of iron overload is definitive, and the likelihood of secondary iron overload is high, based on the following factors: the patient had no family history of primary iron overload; the patient had a documented history of iron infusion before onset, with a cumulative dose of 7500 mg of iron as iron isomaltoside; the patient developed skin pigmentation following iron infusion; MRI results indicated hepatic and splenic iron overload; according to the Ganzoni formula,^[13]^ the patient’s calculated iron requirement was 988 mg. The formula is expressed as follows:


$$\begin{array}{lll} & Body\;weight\;\left(kg\right)\times \left(Target\;hemoglobin-Actual\;hemoglobin\right)\;\left(g/L\right)\; & \times 0.24+Iron\;for\;iron\;stores\;\left(mg\right). \end{array}$$

In this case, using a body weight of 55 kg, a target hemoglobin of 120 g/L, an actual hemoglobin of 83 g/L, and an additional 500 mg of iron for iron stores, the total iron requirement was calculated as: 55 × (120−83) × 0.24 + 500 = 988 mg. However, the actual dose administered was 7500 mg, which is 7.6 times the recommended amount. Iron overload can lead to serious complications, including arthritis, liver fibrosis, cirrhosis, primary liver cancer, and diabetes.^[14]^ For patients with secondary iron overload, therapeutic options include phlebotomy and chelation therapy with agents such as deferoxamine, deferasirox, or deferiprone. Following the diagnosis of iron overload, the patient was referred to the hematology department for consultation, and deferasirox dispersible tablets were initiated.

Low-to-moderate doses of oral iron supplementation remain the first-line therapy for uncomplicated iron deficiency.^[15]^ For certain indications, such as intolerance or inadequate response to oral iron, the need for rapid iron replenishment, or the presence of malabsorption disorders such as allergic enteritis and atrophic gastritis, IV iron administration is recommended.^[16]^ Irrespective of whether oral or IV iron supplements are used, improper administration can lead to iron overload.^[3,17]^ Excessive iron intake may result in acute iron poisoning, with serum iron levels exceeding 90 μmol/L. Nevertheless, case reports have suggested that these instances are infrequently associated with subsequent toxic effects.^[18]^ Monitoring serum iron is critically important to prevent iron poisoning, manage potential hypersensitivity reactions, and assess the effectiveness of treatment. The Ganzoni formula has several limitations and may not adequately restore iron stores in most patients.^[19]^ These limitations arise from its reliance on a fixed target Hb value and its failure to account for the dynamic fluctuations in iron reserves. Consequently, its application should be accompanied by a thorough assessment of the clinical context and iron metabolism markers. In this case, a patient with postoperative blood loss anemia underwent gastrointestinal surgery and received IV iron supplementation. However, the total required iron dose was not accurately calculated post-surgery, and iron metabolism markers were not monitored, resulting in iron overload caused by the inappropriate administration of iron isomaltoside therapy.

Methods for evaluating iron overload include SF testing, liver iron concentration measurement, and MRI. Although SF is commonly used to estimate total body iron levels, it can be influenced by factors such as inflammation, infection, liver dysfunction, and oxidative stress. Liver iron concentration provides a direct reflection of the body’s iron overload status, but its measurement typically requires a liver biopsy, which is associated with limitations including sampling errors, invasiveness, complications, and variability between observers and within samples due to the small sample size.^[20]^ MRI, which is highly sensitive to tissue iron, is increasingly being used as a noninvasive alternative to biopsy for detecting, grading the severity, and monitoring treatment in patients with known or suspected iron overload.^[21]^

IV iron therapy has gained significant popularity owing to the effectiveness of modern formulations, which are generally well-tolerated and do not pose concerns regarding absorption rates or gastrointestinal side effects commonly associated with oral therapies. This has led to a paradigm shift in iron therapy, resulting in a steady rise in prescriptions for new, high-dose preparations. Although IV iron infusions are feasible for the majority of patients, these treatments carry a small risk of severe infusion reactions.^[22,23]^ Iron isomaltoside is a linear structure composed of repeated α1 to 6-linked glucose units, with an average size of 5.2 glucose units and a molecular weight of 1 kDa.^[24]^ This facilitates the gradual release of bioavailable iron into iron-binding proteins.^[25]^ A substantial body of clinical evidence demonstrates that iron isomaltoside is well-tolerated in the treatment of iron deficiency and IDA across various patient populations and therapeutic indications, allowing for high-dose injections within a short time frame.^[26–29]^ Moreover, compared to other IV iron formulations, such as ferric carboxymaltose, iron isomaltoside exhibits superior safety, with a lower risk of severe hypersensitivity reactions and hypophosphatemia.^[30,31]^ As previously noted, the high-dose absorption capacity and sustained-release properties of iron isomaltoside may lead clinicians to overlook associated complications, such as iron overload. Additionally, studies have indicated that iron isomaltoside can induce nitrosative stress, oxidative stress, and apoptosis, which represent significant risks requiring attention.^[32]^

This report focuses on secondary iron overload resulting from iron isomaltoside administration. This iatrogenic condition typically develops within a short timeframe after repeated excessive dosing. To prevent overdose and mitigate the risk of complications, it is crucial to monitor iron metabolism parameters regularly and perform timely evaluations before and after iron therapy. A literature review, including PubMed and other databases, did not identify prior cases of iron overload specifically from iron isomaltoside.

The primary limitations of this study arise from its descriptive research design and single-case nature, both of which constrain the generalizability of the findings. Additionally, because the patient declined a cardiac MRI, it was not possible to determine whether iron deposition was present in the heart.

## 6. Conclusion

Patients receiving IV iron isomaltoside are at risk of iron overload and secondary hemosiderosis. Ferritin and transferrin saturation should be monitored before each dose, particularly in high-risk patients, such as those with systemic lupus erythematosus or recent blood loss.

## Author contributions

**Conceptualization:** Yao Hu, Min Sun, Lihui Zhong, Yuxi Wu, Yuehua Yan, Yuanbing Xiang.

**Data curation:** Yao Hu, Min Sun, Lihui Zhong, Dan Luo, Yuxi Wu, Yuehua Yan, Yuanbing Xiang.

**Formal analysis:** Yao Hu, Min Sun, Lihui Zhong, Dan Luo, Yuxi Wu.

**Methodology:** Yao Hu, Min Sun, Dan Luo, Yuxi Wu, Yuehua Yan, Yuanbing Xiang.

**Writing – original draft:** Yao Hu, Min Sun.

**Writing – review & editing:** Lihui Zhong.

**Investigation:** Dan Luo, Yuehua Yan, Yuanbing Xiang.
